# Desire for Spiritual Growth: A Neglected Outcome Variable

**DOI:** 10.1093/geroni/igaa057.1992

**Published:** 2020-12-16

**Authors:** Julie Hicks Patrick, Alexandria Ebert, Amy Knepple Carney

**Affiliations:** 1 West Virginia University, Morgantown, West Virginia, United States; 2 University of Wisconsin-Oshkosh, Oshkosh, Wisconsin, United States

## Abstract

Social support facilitates reaching health-related goals, but has rarely been examined in relation to achieving religious/spiritual (R/S) goals. Using data from 300+ adults (M age = 40.3, range 18 to 87 yrs) , we examine the prevalence of R/S goals and the influence of age and social interactions on reaching these goals. Multinomial logistic regressions showed that adults who did not have a goal to be more religious/spiritual reported fewer positive interactions, fewer negative interactions, and were younger than those who continued to work toward their R/S goals. Those who had the intention to become more R/S but were not working toward it were younger than those who persisted. The importance of R/S goals are discussed within the context of other self-improvement goals. Unique aspects of these goals and the ways in which social interactions support achieving these goals are highlighted.

